# Molecular Pathways Underlying Cholesterol Homeostasis

**DOI:** 10.3390/nu10060760

**Published:** 2018-06-13

**Authors:** Milessa Silva Afonso, Roberta Marcondes Machado, Maria Silvia Lavrador, Eder Carlos Rocha Quintao, Kathryn J. Moore, Ana Maria Lottenberg

**Affiliations:** 1 Marc and Ruti Bell Vascular Biology and Disease Program, Leon H. Charney Division of Cardiology, Department of Medicine, New York University School of Medicine, New York, NY 10016, USA; kathryn.Moore@nyumc.org; 2 Laboratorio de Lipides (LIM 10), Hospital das Clínicas HCFMUSP, Faculdade de Medicina, Universidade de Sao Paulo, Sao Paulo, SP 05403-000, Brazil; rmarcondesmachado@yahoo.com.br (R.M.M.); mariasilviaferrari@yahoo.com.br (M.S.L.); equintao@terra.com.br (E.C.R.Q.); ana.lottenberg@hc.fm.usp.br (A.M.L.); 3 Department of Cell Biology, New York University School of Medicine, New York, NY 10016, USA; 4 Faculdade Israelita de Ciências da Saúde, Albert Einstein, São Paulo, SP 05403-000, Brazil

**Keywords:** cholesterol, molecular pathways, cardiovascular disease, cholesterol homeostasis, dietary cholesterol

## Abstract

Cholesterol is an essential molecule that exerts pleiotropic actions. Although its presence is vital to the cell, its excess can be harmful and, therefore, sustaining cholesterol homeostasis is crucial to maintaining proper cellular functioning. It is well documented that high plasma cholesterol concentration increases the risk of atherosclerotic heart disease. In the last decades, several studies have investigated the association of plasma cholesterol concentrations and the risk of cardiovascular diseases as well as the signaling pathways involved in cholesterol homeostasis. Here, we present an overview of several mechanisms involved in intestinal cholesterol absorption, the regulation of cholesterol synthesis and uptake. We also discuss the importance of reverse cholesterol transport and transintestinal cholesterol transport to maintain cholesterol homeostasis and prevent atherosclerosis development. Additionally, we discuss the influence of dietary cholesterol on plasma cholesterol concentration and the new recommendations for cholesterol intake in a context of a healthy dietary pattern.

## 1. Introduction

Cholesterol is an important cellular molecule involved not only in the maintenance of membrane permeability and fluidity, but also in the modulation of transmembrane signaling pathways, as well as in the synthesis of steroid hormones, bile acids and vitamin D [1,2,3]. Although cholesterol plays a fundamental role in a plethora of intracellular mechanisms, it is known that individuals with high plasma cholesterol concentration are at increased risk of atherosclerotic heart disease [4]. According to the American Heart Association [5], around 31.7% of US adults have high levels of LDL-C. Because of the strong association of plasma cholesterol levels and cardiovascular disease risk, numerous cellular, experimental and clinical investigations have been conducted in the last decades, in order to elucidate the pathways involved in cholesterol homeostasis and their impact on atherosclerosis development. The aim of this article is to provide an overview of the molecular mechanisms that underlie cholesterol absorption, balance, biosynthesis, reverse cholesterol transport, and the role of dietary cholesterol on the risk of developing cardiovascular disease.

### Cholesterol Absorption

Cholesterol is synthesized by most cells, although the major site of production of cholesterol is in hepatocytes and enterocytes. It is noteworthy that the intestinal cholesterol pool size is influenced mainly by endogenous sources (800–1000 mg), since the diet contributes only 300–400 mg of cholesterol per day [6]. Several mechanisms are involved in the absorption of cholesterol, such as, solubility and the release of sterols from micelles [7] and a regulatory network of proteins that are responsive to sterol balance. The net cholesterol absorption in the upper small intestine is regulated by Niemann–Pick-C1-like-1 (NPC1L1) protein [8]. Once in the enterocyte, the cholesterol molecule is esterified with a fatty acid in a process mediated by acyl-cholesterol acyl transferase (ACAT2), an enzyme localized in the membrane of the endoplasmic reticulum (ER) [9]. The resulting cholesteryl ester is incorporated into chylomicrons and delivered to the Golgi apparatus for further processing and secretion to the circulation through the thoracic duct [10]. A small amount of free cholesterol is excreted back into the intestinal lumen by apical transporters, such as ATP-binding cassette transporters sub-family G member 5 (ABCG5) and member G 8 (ABCG8) [9,11].

**Figure 1:**
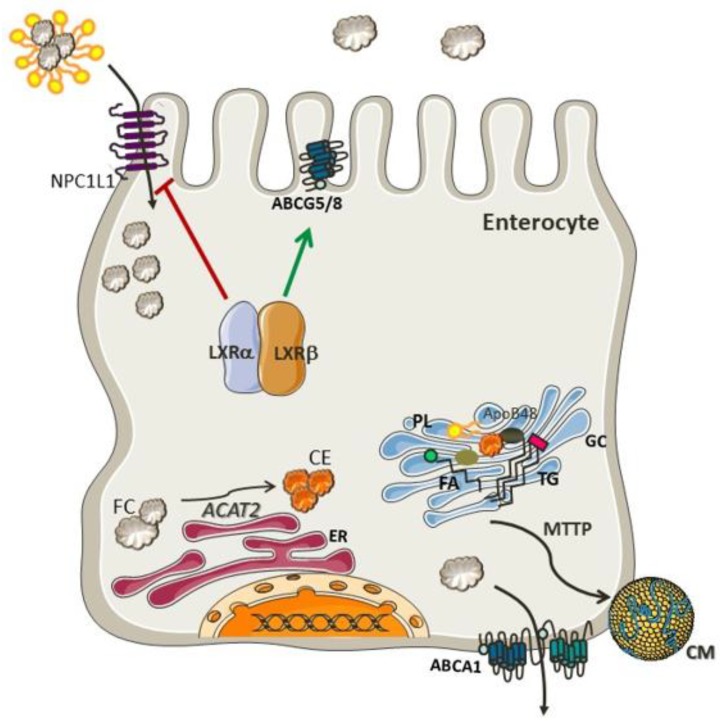
Mechanisms involved in cholesterol absorption. Free cholesterol (FC) transported in the micelles enter the enterocyte through NPC1L1 and is esterified by ACAT2 in the endoplasmic reticulum (ER). Free cholesterol returns to the intestinal lumen via ABCG5/G8 transporters. Cholesteryl ester, triglycerides, and apolipoprotein B-48 (apo B-48), are further processed in the Golgi complex (GC) to form chylomicrons (CM), which are then transferred to the lymphatic system. NPC1L1 and ABCG5/8 expression is controlled by the cholesterol sensors LXRα and LXRβ. Both isoforms inhibit NPC1L1 and activate ABCG5/G8 inducing cholesterol excretion back to the lumen. LXR can also induce ABCA1-mediated cholesterol efflux to prevent cholesterol accumulation in the enterocytes.

The regulation of these processes in intestinal cholesterol uptake and secretion is mediated by Liver X Receptor (LXR), a nuclear receptor involved in the inhibition of NPC1L1 [12] and activation of ABCG5 and ABCG8 [13]. Both isoforms of LXR, LXRα (*Nr1h3*) and LXRβ (*Nr1h2*), act as cholesterol sensors and seem to exert similar effects on the expression of these proteins [14]. Furthermore, LXRs also lead to an increase in cholesterol efflux from enterocytes by increasing the expression of ABCA1 [14]. Altogether, the effects of LXR prevent cholesterol accumulation in the enterocytes. It is important to emphasize that both dietary cholesterol and *de novo* synthesized cholesterol are necessary to maintain intestinal integrity [15]. The molecular pathways involved in cholesterol absorption are portrayed in Figure 1.

## 2. Cholesterol Biosynthesis and Uptake

Cholesterol biosynthesis and uptake are tightly regulated by a mechanism of negative feedback that senses cholesterol and oxysterols. The transcription factor sterol regulatory element-binding protein-2 (SREBP-2; *Srebf2*) is the key regulator of genes involved in cholesterol synthesis such as HMG-CoA reductase (HMGCR), HMG-CoA synthase (HMGCS), and mevalonate kinase (MVK), as well as the LDL receptor (*LDLR*) which is responsible for cholesterol uptake [16,17].

SREBP-2 is attached to the endoplasmic reticulum (ER) membrane and consists of a two-helix transmembrane protein, with the NH2- and COOH-terminal domains facing the cytosol. The NH2-terminal domain comprises the active domain, which once transported to the nucleus, binds to the sterol regulatory element (SRE) and activates the transcription of its target genes. In the ER membrane, SREBP is associated with two proteins, the SREBP cleavage-activating protein (SCAP), which contains eight membrane-spanning regions, and the Insulin-induced gene protein-1 (Insig-1). Upon reduced levels of cholesterol, the SCAP-SREBP complex is dissociated from Insig-1, and the latter undergoes ubiquitination and consequent proteasomal degradation [18]. The complex is then sorted into coat protein II (COPII)-coated vesicles, in a process mediated by a small GTPase, Sar1 [19]. After successive recruitment and clustering of coat proteins, such as Sec23/Sec24 and Sec13/Sec31, COPII coat vesicles are assembled [20] and escort the SCAP-SREBP complex from the ER to the Golgi, where SREBP will be sequentially cleaved by two resident proteases, site-1 (S1P) and site-2 (S2P), releasing the NH2-active domain [17,18,21,22,23]. As a regulatory mechanism, SREBP-2 not only induces the expression of genes involved in cholesterol synthesis and uptake, but it can also bind to the E-box at the ABCA1 promoter region, inhibiting its transcription and, therefore, cholesterol efflux [24]. In addition, miR-33, a microRNA located within an intron in the SREBF2 locus, is co-transcribed with SREBF2 and acts to repress cholesterol trafficking and export, so that the intracellular cholesterol concentrations can be quickly restored [25,26].

In cholesterol replete situations, the Insig1-SCAP-SREBP2 complex is trapped in the ER, as SCAP undergoes several conformational changes that favor its binding to Insulin-induced gene protein-1 (Insig-1). This phenomenon is attributed to the sterol-sensing domain (SSD) in SCAP, which is the cholesterol-dependent binding site for Insig-1 [27]. At the protein level, Insig-1 is stabilized by SCAP, as it prevents Insig-1 ubiquitination and degradation [23]. The presence of cholesterol or 25-hydroxycholesterol in the ER membrane inhibits the binding of coat proteins to SCAP, preventing the assembly of COPII-coated vesicles [19]. This process is regulated by a methionine-glutamate-leucine-alanine-aspartate-leucine (MELADL) sequence present in Loop 6 of SCAP. In fact, it has been demonstrated that the SCAP sequence harbors several domains, which have crucial roles in the trafficking of the SCAP-SREBP complex from the ER to the Golgi [22]. The MELADL sequence at Loop 6 contains a binding site for coat proteins [19]. Therefore, the assembly of COPII-coated vesicles is impaired during cholesterol loading because of the conformational change in SCAP mediated by Insig-1 binding, which displaces the MELADL sequence and makes the binding site unreachable. At the same time, the binding of cholesterol to the luminal loop 1 displaces its binding to loop 7, which confers an open conformation to SCAP, facilitating Insig-1-SCAP binding and preventing the access of COPII proteins to the MELADL sequence, which keeps the SCAP-SREBP-2 complex trapped to the ER [17,23]. It was recently shown that the transcriptional activity of SREBP2 can also be repressed by the ubiquitin E3 ligase, Rnf145 [28]. This LXR target gene induces SCAP ubiquitination on two residues of lysine close to COPII-vesicle binding site, blocking the transport of SCAP-SREBP-2 to the Golgi [28,29]. This illustrates the crosstalk between SREBP and LXR, the master regulators of cholesterol synthesis and efflux, in an attempt to maintain cholesterol homeostasis.

## 3. Cholesterol Synthesis and Enzymatic Control

Cholesterol biosynthesis occurs in the ER and requires more than 30 chemical reactions, with acetate being its precursor [30]. The first two reversible reactions, catalyzed by thiolase and HMG-CoA synthase, lead to the condensation of two molecules of acetate to form acetoatetyl-CoA which is condensed with a third molecule of acetate forming 3-hydroxy-3methylglutaryl-CoA coenzyme A (HMG-CoA). The reaction that follows is the key point of regulation in cholesterol synthesis and is catalyzed by the enzyme HMG-CoA reductase, a transmembrane protein of the ER, which reduces HMG-CoA to mevalonate [30,31]. In consecutive reactions mevalonate is converted into isopentenyl pyrophosphate, an activated isoprene. The next phase promotes the condensation of six molecules of isopentenyl pyrophosphate to form squalene, which is then cyclized and converted to cholesterol through several steps [32].

The transcription of HMG-CoA reductase, which is the rate-limiting enzyme in cholesterol synthesis, is coordinated by SREBP-2, and thus, its expression is governed by cellular cholesterol concentration. HMG-CoA reductase presents a transmembrane sterol-sensing domain (SSD), which shares high homology with the SSD presented in SCAP [33]. As described above, this domain is crucial for the binding of Insig-1 or Insig-2, proteins that are necessary for enzyme degradation and its regulatory control [34]. Insig is constitutively associated with the membrane-bound ubiquitin ligases gp78 and Tcr8 in the ER membrane, and in response to high cholesterol concentration, recruits HMG-CoA reductase [35]. The ubiquitination promoted by gp78 and Tcr8 allows the recognition of HMG-CoA reductase by an ATPase known as vasolin-containing protein (VCP or p97) that induces the proteasomal degradation of the reductase in a sterol-Insig dependent manner [34,36,37,38]. Oxysterols, such as 24-, 25-, and 27-hydroxycholesterol [39], can also induce HMG-CoA reductase ubiquitination mediated by Insig-1 [36,38]. Furthermore, the binding of oxysterols to Insig-1 blocks the transport of SCAP-SREBP-2 to the Golgi, and consequently the translocation of the transcription factor to the nucleus [40]. Therefore, oxysterols reduce HMG-CoA reductase at the mRNA and protein levels, reducing cholesterol synthesis.

Intermediates in cholesterol synthesis, such as lanosterol and 24,25-dehydrolanosterol induces HMG-CoA reductase ubiquitination and degradation in an Insig-dependent manner, without blocking the processing of SREBP-2 [41,42,43]. The same effect was observed for other intermediates, such as 27-hydroxylanosterol, 7-Keto, 25-hydroxycholesterol and 27-hydroxycholesterol [41].

Statins, a class of drugs used to lower plasma cholesterol concentrations, are selective inhibitors of HMG-CoA reductase activity, thus inhibiting endogenous cholesterol synthesis [44]. As a result, LDLR is upregulated in hepatocytes, increasing the uptake of LDL-c particles and reducing plasma cholesterol concentrations [45]. The beneficial use of statins to lower LDL-c plasma concentrations in patients with previous CVD has long been documented and a meta-analysis from the Cochrane Library for the use of statins in primary prevention also found reductions in all-cause mortality and major vascular events among people without evidence of CVD [46,47].

Another point of control on cholesterol synthesis occurs in a later step catalyzed by squalene monooxygenase (SM), enzyme involved in the conversion of squalene to 2,3(S)-mono-oxidosqualene (MOS), a precursor of lanosterol. SM is integrally associated to the ER membrane [48] and its transcription is also controlled by SREBP-2, which implies that cell cholesterol concentration is important to maintain its stability [49,50]. In vitro studies showed that cholesterol, but not oxysterols, induced SM polyubiquitination and degradation through the proteasomal system. This mechanism was Insig-independent [49] and mediated by an E3 ubiquitin ligase, the membrane-associated RING finger 6 (MARCH6) [50]. It is noteworthy that the conformational changes induced by cholesterol in SM structure are crucial to its cholesterol-dependent degradation, in which the *N*-terminus region play a role [48].

**Figure 2:**
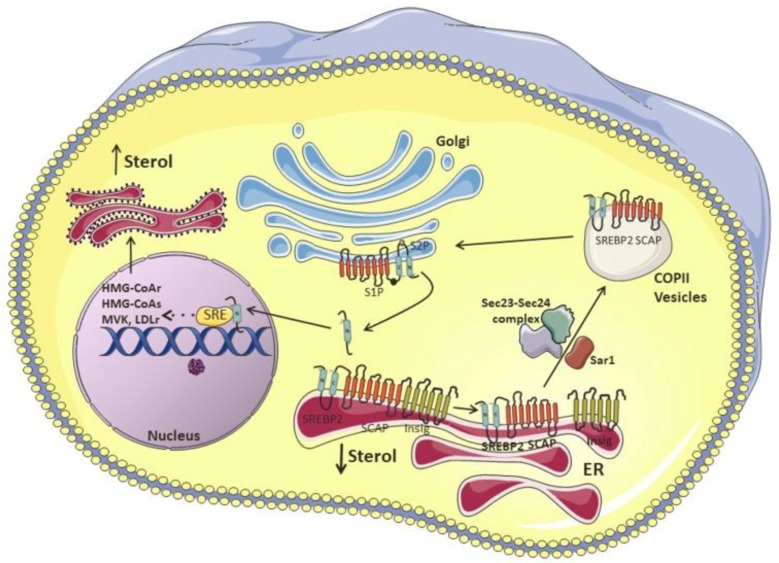
Regulation of cholesterol biosynthesis. The master regulator of cholesterol synthesis and uptake, SREBP-2, is attached to the ER membrane through the interaction with SCAP and Insig-1. In a state of a reduced levels of cholesterol, Insig-1 undergoes proteasomal degradation and the complex SCAP-SREBP is sorted into COPII-coated vesicles, in a process mediated by a small GTPase, Sar1 and the coat proteins Sec23/Sec24 and Sec13/Sec31. COPII coat vesicles escort the SCAP-SREBP complex from ER to Golgi, where SREBP is cleaved by two resident proteases, site-1 (S1P) and site-2 (S2P), releasing its NH2-active domain. In the nucleus, SREBP binds to the SRE inducing the transcription of genes involved in cholesterol synthesis such as HMGCR, HMGS, and MVK, as well as *LDLR* which is responsible for cholesterol uptake, in an attempt to restore the intracellular cholesterol concentrations. ER: endoplasmic reticulum, SREBP-2: sterol regulatory element-binding protein-2; SCAP: SREBP cleavage-activating protein; Insig-1: Insulin-induced gene protein-1.

To summarize, all these regulatory mechanisms of negative feedback help to maintain an adequate cellular cholesterol concentration as shown in Figure 2.

## 4. Cholesterol Balance

There is a close relationship between cholesterol absorption and synthesis that is necessary to maintain whole-body cholesterol balance. Compensatory mechanisms shift the pathways in opposite directions, so that when synthesis rises, absorption decreases and vice versa. As a proof of concept, the treatment of hyperlipidemic men with a cholesterol synthesis inhibitor (Atorvastatin) was associated with a 76% reduction in lathosterol (marker of endogenous cholesterol synthesis) and an increase of 70% in sitosterol (marker of cholesterol absorption). The effects of atorvastatin included the upregulation of genes involved in cholesterol synthesis and uptake (SREBP-2, HMG-CoA reductase, LDLR, and NPC1L1), and downregulation of ABCG5 and ABCG8, which are responsible for the efflux of cholesterol from the enterocytes back to the intestinal lumen [51].

This compensatory mechanism promotes the balance among dietary cholesterol, endogenous synthesis and cholesterol excretion. Several studies using sterol balance techniques investigated the endogenous response to the increase in dietary cholesterol and observed a great variability among participants [52,53]. This variability can be attributed to the alterations in the transcriptional and posttranslational regulation of proteins related to cholesterol efflux and uptake, as well as to proteins involved in cholesterol synthesis.

The increase in dietary cholesterol is compensated by enhanced bile acid synthesis, which is excreted in feces, the main form of cholesterol excretion [54]. In fact, Quintão et al. (1971) [51], demonstrated that the increase in cholesterol intake diminished cholesterol synthesis and led to higher excretion of endogenous cholesterol through the biliary tract. Thus, a reduction in cholesterol synthesis should occur in order to have an effective compensatory mechanism preventing the rise in plasma cholesterol concentration during an increase in cholesterol intake [55].

McNamara et al. (1987) [56], observed that the increase in dietary cholesterol has a marginal effect on total plasma and LDL cholesterol concentrations, due to a decrease on exogenous cholesterol absorption. The increment on dietary cholesterol, from 240 to 800 mg per day, led to a 21% reduction of endogenous cholesterol synthesis, individually estimated by the incorporation of radiolabeled acetate into sterols on peripheral blood mononuclear cells. However, individuals who failed to compensate the increase in cholesterol intake by reducing cholesterol synthesis had a significant increase in plasma cholesterol concentration.

The cholesterol present in the intestinal lumen originates from diet or endogenous sources (bile secretion, TICE and intestinal epithelial sloughing) [57]. In the liver, cholesterol is converted to bile acids, which are conjugated to amino acids (either glycine or taurine), secreted into the bile and further eliminated into the feces. The bile acid secreted into the lumen is reabsorbed in the ileum and returns to the liver in a process called enterohepatic circulation. The bile acid loss in the feces (~5%) is compensated for by its synthesis in the liver in a tightly regulated process, where the bile acids reabsorbed exert a negative feedback on synthesis [58].

## 5. The LDLR and Cholesterol Uptake

The role of the LDLR was depicted by Brown and Goldstein, who demonstrated that defects in LDLR are the cause of the human genetic disease familial hypercholesterolemia (FH) [59]. These patients can inherit one (heterozygosis) or two (homozygosis) mutant copies of the LDL receptor gene. The homozygous form is less common, and its prevalence has been estimated in 1 per 1 million people. In the heterozygous form the frequency is higher, 1:500, and may present even higher prevalence in some populations, ranging from 1:67 to nearly 1:400. In FH homozygotes, plasma cholesterol concentration is very high and atherosclerotic lesions and heart attacks occur early in life [60].

The LDLR is synthesized in the ER and translocates to the cell surface where it can bind with high affinity to LDL particles. The LDLR–LDL particle complex is internalized in coated vesicles through endocytosis. After internalization, the vesicles fuse to one another to form endosomes. Due to an intra-endosomal drop in the pH, the LDLR releases the LDL particle and returns to the cell surface, providing another cycle of endocytosis. Meanwhile the LDL particle is incorporated into lysosomes, where it is degraded [59,60]. The cholesteryl ester is hydrolyzed and remains within the cell, where the free cholesterol can be re-esterified by ACAT and stored in lipid droplets in the cytoplasm or used as a source for the synthesis of steroid hormones or bile acids [60].

It is also known that cholesterol controls expression of the LDLR by a negative feedback mechanism, involving SREBP-2. Therefore, when intracellular cholesterol concentrations are high, the transcription of LDLR is repressed. On the other hand, when cells are cholesterol deprived the transcription of LDLR is induced to promote the uptake of LDL particles from the plasma, increasing intracellular cholesterol concentration [17].

Although all patients with FH have a non-functional LDLR and therefore high LDL cholesterol plasma concentrations, the defects can be ascribed to different mechanisms, that can be divided into four classes: (I) no receptor synthesis, due to a major deletion in the gene; (II) the receptor is synthesized, but the transport from the ER to the Golgi is very slow, and in most cases, it is degraded in the ER, and appearance of the receptor on the cell surface is rare; (III) the receptor is synthesized and reaches the cell surface, but has reduced ability to bind LDL particles; (IV) the receptor fails to cluster in coated pits.

Additional regulatory control of LDLR content at the plasma membrane is performed by proprotein convertase subtilisin/kexin type 9 (PCSK9), which binds to the LDLR directing it to lysosomes where it will be degraded instead of returning to the cell surface [61]. It was demonstrated that mutations with gain of function in the PCSK9 gene are also a form of autosomal-dominant hypercholesterolemia [62].

A very elegant study demonstrated that cells incubated with LXR ligands presented a reduction in the binding and uptake of LDL particles. The mechanism behind it was attributed to the inducible degrader of LDLR (IDOL), which targets the LDLR for ubiquitination [63]. IDOL contains a FERM-domain which interacts with cytoplasmic residues of LDLR triggering its ubiquitination and degradation. Other targets for IDOL include VLDLR and ApoER2 (also known as LRP8), which share homology with LDLR [64].

It is well known that LDL particles are important contributors to atherosclerotic lesion progression [65]. This is mainly attributed to their retention and consequent enzymatic or oxidative modifications in the arterial intima [66]. This process generates aldehydes and ketones that covalently modify ε-amino groups of lysine residues of the protein moiety, [66]. Oxidized LDL (oxLDL) contributes to atherogenesis by delivering cholesterol to macrophages in the arterial wall through Lectin-like oxidized low-density lipoprotein (LDL) receptor-1 (LOX-1), Cluster of Differentiation-36 (CD36), Scavenger receptor class A (SR-A) and Scavenger receptor class B type 1 (SR-B1) culminating in the formation of lipid-laden foam cells that are retained within the arterial wall [67]. To prevent the progression of atherosclerosis, excess cholesterol must be transferred outside the cell in a process called cholesterol efflux [68]. The movement of cholesterol from peripheral tissues to the liver for excretion into the bile, either as bile acid or free cholesterol, is known as reverse cholesterol transport (RCT). In this process, originally described by Glomset et al. (1968) [69], HDL plays a central role, which confers the anti-atherogenic properties to this particle. HDL bears a strong inverse correlation with CVD in epidemiological studies [70], which is thought to be due to its role in RCT.

## 6. Reverse Cholesterol Transport

RCT is the major route for cholesterol removal, and therefore is an essential mechanism to maintain cellular cholesterol homeostasis. Cholesterol efflux from macrophages can occur through a unidirectional ATP-dependent pathway mediated by ABCA1 and/or ABCG1, as well as by SR-BI, in a bidirectional ATP-independent pathway. The efflux of cholesterol can also occur through a receptor-independent diffusion of cholesterol in a gradient dependent process, which contributes to 30% of total cholesterol efflux [71].

Upon cholesterol loading, proteins involved in RCT are upregulated, particularly in response to oxysterols present in oxLDL. Produced intracellularly from cholesterol, the most important endogenous oxysterols are 24(S),25-epoxycholesterol, 24(S)-hydroxycholesterol, 22(R)-hydroxycholesterol, 20(S)-hydroxycholesterol, 25-hydroxycholesterol and 27-hydroxycholesterol [72]. These oxysterols are endogenous ligands for LXR α and β, that heterodimerize with retinoid X receptors (RXRs) and interact with a DR-4 sequence in the promoter region of ABCA1 and ABCG1 to induce transcription [73]. In macrophages, oxysterols not only stimulate cholesterol efflux, but also repress pro-inflammatory signaling cascades, enhance efferocytotic capacity, and promote survival—all of which counteract lesion progression [72]. Monzel et al. (2017) [74] observed that 24-hydroxycholesterol can also directly induce ABCA1/G1 expression. The upregulation of ABCA1 allows the initiation of reverse cholesterol transport, mediating the efflux of free cholesterol and phospholipids from arterial macrophages to the extracellular acceptor apoA-I [75,76].

The structure of human ABCA1 comprises one polypeptide chain, with two nucleotide binding domains (TMD1 and TMD2), each one containing six transmembrane helices. ApoA-I binds to two extracellular domains (ECD1 and ECD2), that together enclose a hydrophobic tunnel working as a passage for lipids to be exported to this extracellular acceptor [77]. ABCA1 and apoA-I form a high-affinity molecular complex required for optimal cholesterol efflux [78]. Moreover, this interaction extends the half-life of ABCA1 in the plasma membrane, as it promotes the dephosphorylation of ABCA1 in its PEST (proline, glutamic acid, serine and threonine) sequence, which is important to avoid its degradation by calpains [79].

After being removed from cells, free cholesterol present in the discoidal lipid-poor apo A-I is esterified to an acyl chain, forming cholesterol ester through lecithin:cholesterol acyltransferase (LCAT). The LCAT-mediated cholesterol esterification consists of several steps, starting with the enzyme binding to the lipoprotein/lipid surface with the help of nonpolar amino acids in the membrane-binding domain [80], until the transfer of the acyl group to cholesterol to complete the esterification process [81,82].

The central domain of LCAT comprises the catalytic triad Asp345, His377 and Ser181 [83], which is required for the α/β hydrolase catalytic activity. The crystal structure of the LCAT has an α/β hydrolase core with two additional subdomains required for interfacial activation and contains the lid and amino acids that shape the substrate binding pocket [84]. The lid-loop presented in the LCAT can move apart from the opening tunnel sending lipids to the active site, where catalytic triad is located and additionally, FC molecules are attracted adjacent to the tunnel [80,84]. This process culminates in the formation of larger spherical mature particles, such as HDL3 and finally HDL2.

Mature HDL particles promote cholesterol efflux from macrophages through ABCG1 [85] and scavenger receptor class B type I (SR-BI). Another important role of ABCG1 is to promote the efflux of the pro-apoptotic oxysterol 7-ketocholesterol (7KC) from peripheral cells [85], through AMPK activation [86]. This is an important function as 7-KC, 7α and 7β-hydroxycholesterol, products of non-enzymatic cholesterol oxidation, together with 27-hydroxycholesterol (produced via cholesterol 27-hydroxilase, CYP27A1), are the major oxysterols found in atherosclerotic lesions [72]. On this note, the ω-carboxyl group in 7-KC-9-carboxynonanoate (oxLig-1) is an epitope of oxLDL that mediates the binding of oxLDL to LOX-1, leading to the upregulation of ABCA1 through PPARγ-LXRα-ABCA1 signaling pathway [87]. Moreover, the binding of oxLDL to CD36 after oxLig-1 stimulation, upregulates caveolin-1 and promote cholesterol efflux in macrophages, in a process mediated by Src-JNK/ERK1/2-NF-κB signal transduction [87]. Caveolin-1 is the main structural protein of caveolae and is involved in intracellular cholesterol trafficking and removal [88].

The major sources of apoA-I are liver and intestine [89]. However, during inflammation, moderate expression of apoA-I can be upregulated in macrophage and human monocytes in a TNFα-dependent manner through NFκB, MEK1/2, JNK, and MAPKp38 signaling pathways [90,91]. More recently, Shavva et al. (2018) [91] demonstrated that TNFα regulates the binding of PPARα, LXRα, and LXRβ to the apoA-I promoter region in macrophages during inflammation [91].

In vitro study showed that the activation of TLR2/4 upregulates ABCA1 expression, while also downregulating ABCG1. This may be ascribed by an interplay of several pathways such as TLR/MYD88/LXR, that enhances ABCA1 transcription; the NF-κB pathway, that downregulates the ABCG1 mRNA; and the p38 pathway that enhances and stabilizes ABCA1 mRNA. As a result, the crosstalk increases the ABCA1 mRNA but not the protein content, while leading to a down-regulation of the ABCG1 mRNA and an increase ABCG1 protein [92].

After removing the cholesterol from peripheral tissues, HDL returns to the liver and the content of triglycerides and phospholipids are hydrolyzed by hepatic lipase, resulting in the assembly of small HDL which is taken up by SR-BI present in the liver [93]. An alternative route to deliver cholesterol to the liver involves cholesteryl ester transfer protein (CETP), which mediates bidirectional transfer of cholesteryl esters (CE) and triglycerides (TG) between plasma lipoproteins [94]. Therefore, CETP transfers cholesterol from HDL to the apo B rich particles that will be further delivered to the liver through B/E receptor [95].

CETP is a member of the lipid transfer protein/lipopolysaccharide binding protein (LTP/LBP) gene family [96] and its expression and activity are upregulated under dietary and plasma cholesterol stimuli [97,98]. Due to its high flexibility, CETP undertakes a rotation movement when it binds to neutral lipids. This motion enables CETP to bind to the surface of lipoproteins that vary widely in size and surface curvature [94]. The CETP structure presents a hydrophobic tunnel with openings in the N- and C-terminal domains, where phospholipids are accommodated. The amphiphilic property of phospholipids increases the protein compatibility with an aqueous milieu [99]. Therefore, CETP can perform lipid exchange among lipoproteins, because both ends of the tunnel can accept neutral lipids from a donor lipoprotein particle and deliver to an acceptor [99].

Finally, cholesterol is also delivered to the liver by apo B-rich particles to the LDLR. Esterified cholesterol will be hydrolyzed by cholesteryl ester hydrolase and excreted into bile [100]. The rate-limiting enzyme of bile acid synthesis is cholesterol 7α-hydroxylase (Cyp7A1) [101], while Cyp8b1, is required for cholic acid synthesis [102]. Bile acids can be synthesized through classical and/or alternative pathways. In the classical pathway, Cyp7a1 plays a central role by placing a hydroxyl group to the carbon 7 of the cholesterol for further processing [103]. The transcription of Cyp7a1 is stimulated by the LXR/RXR pathway in a manner dependent on the concentration of hepatic cholesterol [104,105]. Since high levels of bile acids can be toxic and result in severe metabolic complications, a feedback mechanism to inhibit their synthesis is necessary. In this case, the excess of hepatic bile acids decreases Cyp7a1 expression through the transcriptional repressor small heterodimer partner 1 (SHP-1/*Nr0b2*), in a process mediated by FXR [106,107]. Upon activation, FXR also induces the transcription of the RNA-binding protein Zfp36l1, which binds of the 3′-UTR of Cyp7a1 inducing its degradation [106].

An alternative pathway for cholesterol excretion is the non-biliary route, called the trans-intestinal cholesterol excretion (TICE), which contributes significantly to neutral sterol excretion in humans [108]. The cholesterol uptake from plasma lipoproteins by enterocytes at the basolateral membrane together with cholesterol excretion by ABCG5/G8 are the main routes of TICE. The most recognized aspect regarding intestinal cholesterol uptake relates with the presence of LDLR localized on the basolateral side of enterocytes.

**Figure 3:**
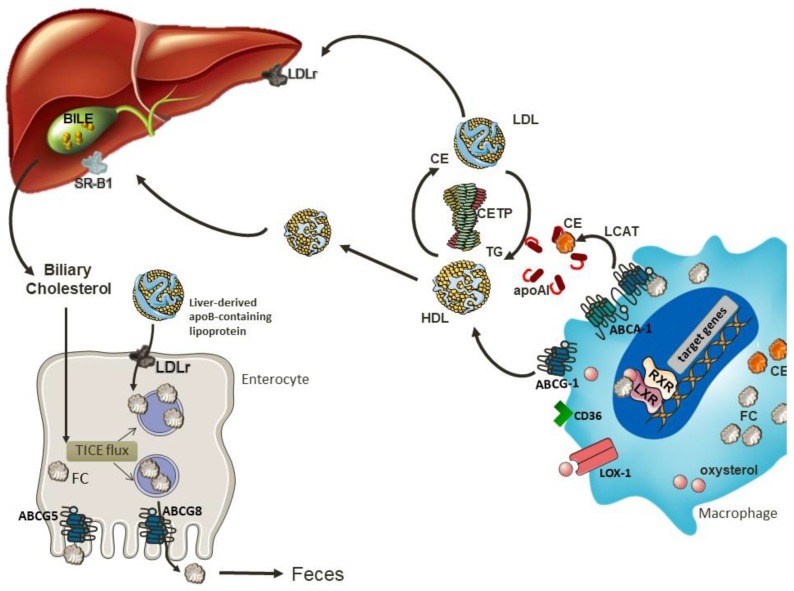
The regulatory pathways of reverse cholesterol transport: oxLDL contributes to atherogenesis by delivering cholesterol and oxysterol to macrophages in a process mediated by scavenger receptors (CD36, SR-A, SR-BI and LOX-1) present on the surface of these cells. Oxysterols are strong ligands of LXR, which heterodimerizes with RXR, and therefore induces ABCA1 and ABCG1 expression. ABCA1 promotes cholesterol efflux to apoA-I, while ABCG1 removes cholesterol from cells by interacting with HDL. The FC present in the discoidal lipid-poor apoA-I is esterified to an acyl chain through LCAT. The cholesterol acceptors apoA-I and HDL drive cholesterol from the periphery to the liver for bile secretion. HDL delivers cholesterol to the liver after partial hydrolysis of its triglycerides and phospholipids content by hepatic lipase, resulting in a smaller particle that is taken up by SR-BI. Cholesterol can also be delivered to the liver through B/E receptor in a process mediated by CETP, which transfers cholesterol from HDL to the apo B rich particles. In the liver, cholesterol is converted into bile acids and secreted in to the bile. Cholesterol from plasma lipoproteins can also be taken up by enterocytes at the basolateral membrane and subsequently excreted into the lumen by ABCG5/G8 in an alternative, non-biliary route known as TICE. FC: free cholesterol; CE: cholesterol ester; TG: triglycerides; CETP: cholesteryl ester transfer protein; LCAT: lecithin: cholesterol acyltransferase; TICE: trans-intestinal cholesterol excretion.

Some studies have investigated the role of HDL in this process and the results seem to be controversial [109,110]. A study performed in mice after bile diversion and intestinal cannulation showed that both HDL and LDL deliver cholesterol for TICE at the jejunal basolateral membrane [109]. In this investigation, TICE activity was increased by statins and decreased by PCSK9, a protein involved in LDLR degradation, emphasizing the relevance of this receptor to TICE. However, TICE is preserved in *Ldlr*^−/−^ mice, which suggests the presence of an alternative pathway [109]. A study conducted in *Srb1*^−/−^ mice, a model characterized by high HDL-C levels, showed a two-fold increase in TICE compared to wild type mice [111]. Subsequently, the same group decided to inject HDL containing radiolabeled cholesterol intravenously into WT and *Abca1*^−/−^*Srb1*^−/−^ mice and they observed that TICE was not regulated by HDL, as the intestine of both mice strains did not present any difference in cholesterol uptake or secretion [110]. On this note, the role of cholesterol uptake from enterocytes at the basolateral membrane remains to be elucidated. The mechanisms involved in reverse cholesterol transport and trans-intestinal cholesterol excretion are shown in Figure 3.

## 7. Conditions that Impair the Efficiency of the RCT

The composition of HDL is linked to its efficiency in promoting the removal of cholesterol from macrophages, and consequently the sterol excretion through a biliary route. The determinants involved in HDL functionality are concentration, size and composition of the particle, together with gender, body mass index (BMI), age and lifestyle [112].

Several studies have demonstrated that inflammation impairs several steps of RCT. An in vitro study showed that LPS impaired cholesterol efflux of primary human monocyte-derived macrophages to apoA-I and serum, and simultaneously reduced ABCA1 expression [113]. In the same direction, during experimental endotoxemia, a reduced flux of ^3^H-cholesterol from macrophages to plasma, HDL fractions, bile and feces was observed [113]. Moreover, the consumption of a high fat diet enriched in saturated fatty acids increased the concentrations of the acute phase proteins SAA1, SAA2, hemopexin and haptoglobin in small HDL particles. Concomitant with this alteration in the HDL proteomic profile, ABCG5 expression was reduced in the liver, indicating that the levels of acute phase protein on HDL may be a novel biomarker of impaired liver-to feces RCT in vivo [114].

It is well known that diabetes mellitus exacerbates the risk for cardiovascular disease in a mechanism partially dependent on the impairment of ABC transporter functionality. Studies have clearly demonstrated that advanced glycated albumin, which is prevalent in diabetes, disturbs reverse cholesterol transport through cellular mechanisms involving induction of ER stress, production of reactive oxygen species and reprogramming of gene expression towards a pro-inflammatory phenotype [115,116,117]. It was recently demonstrated that advanced glycated end products (AGE) degrade ABCA1 through the proteasomal and lysosomal systems [118]. In this study, the authors observed that knockdown of RAGE, the receptor for AGEs, prevented the reduction in ABCA1. In fact, Daffu et al. (2015) [119] demonstrated that AGEs reduce ABCG1 transcription by repressing the recruitment of PPARG to its promoter region. Together, these mechanisms explain how AGEs lead to macrophage lipid accumulation and contribute to atherosclerosis progression.

The post-translational alterations in ABC transporters were also reported in CHO cells overexpressing ABC transporters. Aleidi et al. demonstrated that the E3 ubiquitin ligases HUWE1 (HECT, UBA, and WWE domain containing 1, E3 ubiquitin protein ligase) and NEDD4-1 (Neural precursor cell-expressed developmentally down regulated gene 4) were associated to the degradation of ABCG1 [120], meanwhile HETC was involved in ABCA1 ubiquitination [121].

## 8. New Recommendations on Dietary Cholesterol

In recent years, the role of dietary cholesterol in the incidence of complications attributed to atherosclerosis has been questioned in such a way that the American Heart Association has ceased to consider limiting the intake of eggs for protection against cardiovascular atherosclerotic disease (CHD). In this sense, the 2015–2020 Dietary Guidelines for Americans have removed the recommendation of limiting cholesterol intake to no more than 300 mg per day [122]. However, they do suggest that dietary cholesterol remains important to consider in order to build healthy eating patterns [123]. In fact, it is emphasized that the dietary cholesterol intake should be as little as possible, as recommended by the Institute of Medicine [123]. Generally, dietary sources containing high amounts of cholesterol are also rich in saturated fatty acids, such as fatty meats and high-fat dairy products. The USDA Food Patterns focus on limiting saturated fats to less than 10% per day, which should be sufficient to limit the cholesterol intake as well.

This conclusion has been supported by some but not all investigations. Accordingly, in an investigation in Finland the intake of eggs or cholesterol does not associate with increased risk for cardiovascular disease (CVD) [124]. Another investigation in the US concludes that low or moderate egg intake is inversely related to the severity of the atherosclerotic lesion in the carotids [125]. On the other hand, although the same findings were reported in the population of Korea [126], a correlation between the consumption of eggs and coronary artery calcification was reported [127].

The importance of dietary cholesterol in CVD continues to be discussed as shown in a recent review in which the authors concluded that although eggs constitute a main source of cholesterol, their role in CVD is tenuous [128]. In support of their conclusion, a meta-analysis reported that egg consumption does not associate with the risk to CVD in the general population, although the authors emphasize that excessive egg consumption increases the incidence of type 2 diabetes among some of the general population and CVD comorbidity among diabetic patients [129].

Nonetheless, the combination of modifiable lifestyle risk factors seems to account for less than 40% of CHD mortality, and consuming one egg a day accounts for less than 1% of CHD risk. Hence, focusing on decreasing egg intake as an approach to modifying CHD risk would be expected to yield minimal results as compared to changing other behaviors such as smoking [130].

This conclusion is supported in at least a systematic review based on 40 studies involving 361,923 participants suggesting that previous populational studies were heterogeneous and lacked the methodologic rigor to draw conclusions regarding the effects of dietary cholesterol on CVD risk [32]. Nonetheless, the latter review showed that the concentration of LDL-C increases in plasma when the daily intake of cholesterol was less than 900 mg, while the elevation of HDL-C, which has anti-atherogenic effects, occurred only in the range from 650 to 900 mg/day, and not when below 650 mg/day. In other words, this analysis brings to light the fact that the cholesterol consumed by most of the population, which is typically between 250 and 350 mg/day, elicits a pro-atherogenic lipoprotein profile that contrasts to the conclusion of a recent revision that admits very discreet variation in the LDL-C/HDL-C plasma ratio due to cholesterol intake [128].

Furthermore, it has been described that intestinal absorption of cholesterol varies between individuals, with some, but not all showing a rise in blood cholesterol concentrations [131]. It has also been reported that intestinal absorption of cholesterol is increased by approximately 40% in cases of familial autosomal dominant hypercholesterolemia and no mutations in the low-density lipoprotein or apolipoprotein B gene [132]. Consequently, both the latter cases and the hyper-responders to dietary cholesterol should benefit from the risk to CHD by drastic reduction on alimentary cholesterol and the blockage of its absorption by phytosterols and other drugs, such as ezetimibe. Such cases constitute a minority in the population so that they do not represent a tangible impact in population research on the role of dietary cholesterol absorption in CHD, however, they need to be identified when treating them with a view to more efficient reduction of blood cholesterol.

It is important to take into account that the intake of cholesterol is just one of several food components that influence the concentration of plasma cholesterol, such as the type of fatty acid ingested, the amount of fibers, and the total amount of calories that together should be modified to get benefit in cardiovascular disease [133]. In fact, simultaneous publications emphasize that it is the association of food interventions that confers the recipe for protection against cardiovascular disease [134], in agreement with AHA and American Dietary Guidelines that highlights the relevance of healthy dietary patterns.

## References

[1] Ikonen E. (2008). Cellular cholesterol trafficking and compartmentalization. Nat. Rev. Mol. Cell Biol..

[2] Lingwood D., Simons K. (2010). Lipid rafts as a membrane-organizing principle. Science.

[3] Maxfield F.R., van Meer G. (2010). Cholesterol, the central lipid of mammalian cells. Curr. Opin. Cell Biol..

[4] Bandyopadhyay D., Ashish K., Hajra A., Qureshi A., Ghosh R.K. (2018). Cardiovascular Outcomes of PCSK9 Inhibitors: With Special Emphasis on Its Effect beyond LDL-Cholesterol Lowering. J. Lipids.

[5] Benjamin E.J., Virani S.S., Callaway C.W., Chamberlain A.M., Chang A.R., Cheng S., Chiuve S.E., Cushman M., Delling F.N., Deo R. (2018). Heart Disease and Stroke Statistics-2018 Update: A Report from the American Heart Association. Circulation.

[6] Mok H.Y., von Bergmann K., Grundy S.M. (1979). Effects of continuous and intermittent feeding on biliary lipid outputs in man: Application for measurements of intestinal absorption of cholesterol and bile acids. J. Lipid Res..

[7] Ikeda I. (2015). Factors affecting intestinal absorption of cholesterol and plant sterols and stanols. J. Oleo Sci..

[8] Altmann S.W., Davis H.R., Zhu L.J., Yao X., Hoos L.M., Tetzloff G., Iyer S.P., Maguire M., Golovko A., Zeng M. (2004). Niemann-Pick C1 Like 1 protein is critical for intestinal cholesterol absorption. Science.

[9] Nguyen T.M., Sawyer J.K., Kelley K.L., Davis M.A., Rudel L.L. (2012). Cholesterol esterification by ACAT2 is essential for efficient intestinal cholesterol absorption: Evidence from thoracic lymph duct cannulation. J. Lipid Res..

[10] Iqbal J., Hussain M.M. (2009). Intestinal lipid absorption. Am. J. Physiol. Endocrinol. Metab..

[11] Yu L., Hammer R.E., Li-Hawkins J., Von Bergmann K., Lutjohann D., Cohen J.C., Hobbs H.H. (2002). Disruption of Abcg5 and Abcg8 in mice reveals their crucial role in biliary cholesterol secretion. Proc. Natl. Acad. Sci. USA.

[12] Sugizaki T., Watanabe M., Horai Y., Kaneko-Iwasaki N., Arita E., Miyazaki T., Morimoto K., Honda A., Irie J., Itoh H. (2014). The Niemann-Pick C1 like 1 (NPC1L1) inhibitor ezetimibe improves metabolic disease via decreased liver X receptor (LXR) activity in liver of obese male mice. Endocrinology.

[13] Yu L., York J., von Bergmann K., Lutjohann D., Cohen J.C., Hobbs H.H. (2003). Stimulation of cholesterol excretion by the liver X receptor agonist requires ATP-binding cassette transporters G5 and G8. J. Biol. Chem..

[14] Hu X., Steffensen K.R., Jiang Z.Y., Parini P., Gustafsson J.Å., Gåfvels M., Eggertsen G. (2012). LXRβ activation increases intestinal cholesterol absorption, leading to an atherogenic lipoprotein profile. J. Intern. Med..

[15] Hui D.Y. (2017). The good side of cholesterol: A requirement for maintenance of intestinal integrity. J. Lipid Res..

[16] Adams C.M., Reitz J., De Brabander J.K., Feramisco J.D., Li L., Brown M.S., Goldstein J.L. (2004). Cholesterol and 25-hydroxycholesterol inhibit activation of SREBPs by different mechanisms, both involving SCAP and Insigs. J. Biol. Chem..

[17] Brown M.S., Radhakrishnan A., Goldstein J.L. (2018). Retrospective on Cholesterol Homeostasis: The Central Role of Scap. Annu. Rev. Biochem..

[18] Gong Y., Lee J.N., Lee P.C.W., Goldstein J.L., Brown M.S., Ye J. (2006). Sterol-regulated ubiquitination and degradation of Insig-1 creates a convergent mechanism for feedback control of cholesterol synthesis and uptake. Cell Metab..

[19] Sun L.-P., Li L., Goldstein J.L., Brown M.S. (2005). Insig required for sterol-mediated inhibition of Scap/SREBP binding to COPII proteins in vitro. J. Biol. Chem..

[20] Sato K. (2004). COPII coat assembly and selective export from the endoplasmic reticulum. J. Biochem..

[21] Sun L.-P., Seemann J., Brown M.S., Goldstein J.L. (2007). Sterol-regulated transport of SREBPs from endoplasmic reticulum to Golgi: Insig renders sorting signal in Scap inaccessible to COPII proteins. Proc. Natl. Acad. Sci. USA.

[22] Brown A.J., Sun L., Feramisco J.D., Brown M.S., Goldstein J.L. (2002). Cholesterol addition to ER membranes alters conformation of SCAP, the SREBP escort protein that regulates cholesterol metabolism. Mol. Cell.

[23] Goldstein J.L., DeBose-Boyd R.A., Brown M.S. (2006). Protein sensors for membrane sterols. Cell.

[24] Zeng L., Liao H., Liu Y., Lee T.S., Zhu M., Wang X., Stemerman M.B., Zhu Y., Shyy J.Y. (2004). Sterol-responsive element-binding protein (SREBP) 2 down-regulates ATP-binding cassette transporter A1 in vascular endothelial cells: A novel role of SREBP in regulating cholesterol metabolism. J. Biol Chem..

[25] Rayner K.J., Sheedy F.J., Esau C.C., Hussain F.N., Temel R.E., Parathath S., van Gils J.M., Rayner A.J., Chang A.N., Suarez Y. (2011). Antagonism of miR-33 in mice promotes reverse cholesterol transport and regression of atherosclerosis. J. Clin. Investig..

[26] Rayner K.J., Suárez Y., Dávalos A., Parathath S., Fitzgerald M.L., Tamehiro N., Fisher E.A., Moore K.J., Fernández-Hernando C. (2010). MiR-33 contributes to the regulation of cholesterol homeostasis. Science.

[27] Feramisco J.D., Radhakrishnan A., Reitz J., Brown M.S., Goldstein J.L. (2005). Intramembrane aspartic acid in SCAP protein governs cholesterol-induced conformational change. Proc. Natl. Acad. Sci. USA.

[28] Cook E.C., Nelson J.K., Sorrentino V., Koenis D., Moeton M., Scheij S., Ottenhoff R., Bleijlevens B., Loregger A., Zelcer N. (2017). Identification of the ER-resident E3 ubiquitin ligase RNF145 as a novel LXR-regulated gene. PLoS ONE.

[29] Zhang L., Rajbhandari P., Priest C., Sandhu J., Wu X., Temel R., Castrillo A., de Aguiar Vallim T.Q., Sallam T., Tontonoz P. (2017). Inhibition of cholesterol biosynthesis through RNF145-dependent ubiquitination of SCAP. eLife.

[30] Alphonse P.A., Jones P.J. (2016). Revisiting Human Cholesterol Synthesis and Absorption: The Reciprocity Paradigm and its Key Regulators. Lipids.

[31] Vaklavas C., Chatzizisis Y.S., Ziakas A., Zamboulis C., Giannoglou G.D. (2009). Molecular basis of statin-associated myopathy. Atherosclerosis.

[32] Berger S., Raman G., Vishwanathan R., Jacques P.F., Johnson E.J. (2015). Dietary cholesterol and cardiovascular disease: A systematic review and meta-analysis. Am. J. Clin. Nutr..

[33] Brown M.S., Goldstein J.L. (2009). Cholesterol feedback: From Schoenheimer’s bottle to Scap’s MELADL. J. Lipid Res..

[34] Sever N., Song B.L., Yabe D., Goldstein J.L., Brown M.S., DeBose-Boyd R.A. (2003). Insig-dependent ubiquitination and degradation of mammalian 3-hydroxy-3-methylglutaryl-CoA reductase stimulated by sterols andgeranylgeraniol. J. Biol. Chem..

[35] Jo Y., Lee P.C., Sguigna P.V., DeBose-Boyd R.A. (2011). Sterol-induced degradation of HMG-CoA reductase depends on interplay of two Insigs and two ubiquitin ligases, gp78 and Trc8. Proc. Natl. Acad. Sci. USA.

[36] Song B.L., Sever N., DeBose-Boyd R.A. (2005). Gp78, a membrane-anchored ubiquitin ligase, associates with Insig-1 and couples sterol-regulated ubiquitination to degradation of HMG CoA reductase. Mol. Cell.

[37] Hartman I.Z., Liu P., Zehmer J.K., Luby-Phelps K., Jo Y., Anderson R.G., DeBose-Boyd R.A. (2010). Sterol-induced dislocation of 3-hydroxy-3-methylglutaryl coenzyme A reductase from endoplasmic reticulum membranes into the cytosol through a subcellular compartment resembling lipid droplets. J. Biol. Chem..

[38] Elsabrouty R., Jo Y., Dinh T.T., DeBose-Boyd R.A. (2013). Sterol-induced dislocation of 3-hydroxy-3-methylglutaryl coenzyme A reductase from membranes of permeabilized cells. Mol. Biol. Cell.

[39] DeBose-Boyd R.A. (2008). Feedback regulation of cholesterol synthesis: Sterol-accelerated ubiquitination and degradation of HMG CoA reductase. Cell Res..

[40] Radhakrishnan A., Ikeda Y., Kwon H.J., Brown M.S., Goldstein J.L. (2007). Sterol-regulated transport of SREBPs from endoplasmic reticulum to Golgi: Oxysterols block transport by binding to Insig. Proc. Natl. Acad. Sci. USA.

[41] Song B.L., Javitt N.B., DeBose-Boyd R.A. (2005). Insig-mediated degradation of HMG CoA reductase stimulated by lanosterol, an intermediate in the synthesis of cholesterol. Cell Metab..

[42] Lange Y., Ory D.S., Ye J., Lanier M.H., Hsu F.F., Steck T.L. (2008). Effectors of rapid homeostatic responses of endoplasmic reticulum cholesterol and 3-hydroxy-3-methylglutaryl-CoA reductase. J. Biol. Chem..

[43] Sharpe L.J., Brown A.J. (2013). Controlling cholesterol synthesis beyond 3-hydroxy-3-methylglutaryl-CoA reductase (HMGCR). J. Biol. Chem..

[44] Sirtori C.R. (2014). The pharmacology of statins. Pharmacol. Res..

[45] Lamon-Fava S. (2013). Statins and lipid metabolism: An update. Curr. Opin. Lipidol..

[46] Scandinavian Simvastatin Study Group (1994). Randomised trial of cholesterol lowering in 4444 patients with coronary heart disease: The Scandinavian Simvastatin Survival Study (4S). Lancet.

[47] Taylor F., Huffman M.D., Macedo A.F., Moore T.H., Burke M., Davey Smith G., Ward K., Ebrahim S. (2013). Statins for the primary prevention of cardiovascular disease. Cochrane Database Syst. Rev..

[48] Howe V., Chua N.K., Stevenson J., Brown A.J. (2015). The Regulatory Domain of Squalene Monooxygenase Contains a Re-entrant Loop and Senses Cholesterol via a Conformational Change. J. Biol. Chem..

[49] Gill S., Stevenson J., Kristiana I., Brown A.J. (2011). Cholesterol-dependent degradation of squalene monooxygenase, a control point in cholesterol synthesis beyond HMG-CoA reductase. Cell Metab..

[50] Zelcer N., Sharpe L.J., Loregger A., Kristiana I., Cook E.C., Phan L., Stevenson J., Brown A.J. (2014). The E3 ubiquitin ligase MARCH6 degrades squalene monooxygenase and affects 3-hydroxy-3-methyl-glutaryl coenzyme A reductase and the cholesterol synthesis pathway. Mol. Cell. Biol..

[51] Tremblay A.J., Lamarche B., Lemelin V., Hoos L., Benjannet S., Seidah N.G., Davis H.R., Couture P. (2011). Atorvastatin increases intestinal expression of NPC1L1 in hyperlipidemic men. J. Lipid Res..

[52] Quintão E., Grundy S.M., Ahrens E.H. (1971). Effects of dietary cholesterol on the regulation of total body cholesterol in man. J. Lipid Res..

[53] Maranhão R.C., Quintão E.C. (1983). Long term steroid metabolism balance studies in subjects on cholesterol-free and cholesterol-rich diets: Comparison between normal and hypercholesterolemic individuals. J. Lipid Res..

[54] Duane W.C. (1993). Effects of lovastatin and dietary cholesterol on sterol homeostasis in healthy human subjects. J. Clin. Investig..

[55] Nestel P.J., Poyser A. (1976). Changes in cholesterol synthesis and excretion when cholesterol intake is increased. Metabolism.

[56] McNamara D.J., Kolb R., Parker T.S., Batwin H., Samuel P., Brown C.D., Ahrens E.H. (1987). Heterogeneity of cholesterol homeostasis in man. Response to changes in dietary fat quality and cholesterol quantity. J. Clin. Investig..

[57] Wang D.Q. (2007). Regulation of intestinal cholesterol absorption. Annu. Rev. Physiol..

[58] Chiang J.Y. (2013). Bile acid metabolism and signaling. Compr. Physiol..

[59] Goldstein J.L., Brown M.S. (1976). The LDL pathway in human fibroblasts: A Receptor-mediated mechanism for the regulation of cholesterol metabolism. Curr. Top. Cell. Regul..

[60] Goldstein J.L., Brown M.S. (2009). The LDL receptor. Arterioscler. Thromb. Vasc. Biol..

[61] Poirier S., Mayer G., Poupon V., McPherson P.S., Desjardins R., Ly K., Asselin M.C., Day R., Duclos F.J., Witmer M. (2009). Dissection of the endogenous cellular pathways of PCSK9-induced low density lipoprotein receptor degradation: Evidence for an intracellular route. J. Biol. Chem..

[62] Abifadel M., Varret M., Rabès J.P., Allard D., Ouguerram K., Devillers M., Cruaud C., Benjannet S., Wickham L., Erlich D. (2003). Mutations in PCSK9 cause autosomal dominant hypercholesterolemia. Nat. Genet..

[63] Zelcer N., Hong C., Boyadjian R., Tontonoz P. (2009). LXR regulates cholesterol uptake through Idol-dependent ubiquitination of the LDL receptor. Science.

[64] Hong C., Duit S., Jalonen P., Out R., Scheer L., Sorrentino V., Boyadjian R., Rodenburg K.W., Foley E., Korhonen L. (2010). The E3 ubiquitin ligase IDOL induces the degradation of the low density lipoprotein receptor family members VLDLR and ApoER2. J. Biol. Chem..

[65] Bhakdi S., Dorweiler B., Kirchmann R., Torzewski J., Weise E., Tranum-Jensen J., Walev I., Wieland E. (1995). On the pathogenesis of atherosclerosis: Enzymatic transformation of human low density lipoprotein to an atherogenic moiety. J. Exp. Med..

[66] Parthasarathy S., Raghavamenon A., Garelnabi M.O., Santanam N. (2010). Oxidized low-density lipoprotein. Methods Mol. Biol..

[67] Arjuman A., Chandra N.C. (2017). LOX-1: A potential target for therapy in atherosclerosis; an in vitro study. Int. J. Biochem. Cell Biol..

[68] Sacks F.M., Jensen M.K. (2018). From High-Density Lipoprotein Cholesterol to Measurements of Function: Prospects for the Development of Tests for High-Density Lipoprotein Functionality in Cardiovascular Disease. Arterioscler. Thromb. Vasc. Biol..

[69] Glomset J.A. (1968). The plasma lecithins:cholesterol acyltransferase reaction. J. Lipid Res..

[70] Karathanasis S.K., Freeman L.A., Gordon S.M., Remaley A.T. (2017). The Changing Face of HDL and the Best Way to Measure It. Clin. Chem..

[71] Brufau G., Groen A.K., Kuipers F. (2011). Reverse cholesterol transport revisited: Contribution of biliary versus intestinal cholesterol excretion. Arterioscler. Thromb. Vasc. Biol..

[72] Olkkonen V.M. (2012). Macrophage oxysterols and their binding proteins: Roles in atherosclerosis. Curr. Opin. Lipidol..

[73] Huwait E.A., Singh N.N., Michael D.R., Davies T.S., Moss J.W., Ramji D.P. (2015). Protein Kinase C is Involved in the Induction of ATP-Binding Cassette Transporter A1 Expression by Liver X Receptor/Retinoid X Receptor Agonist in Human Macrophages. J. Cell. Physiol..

[74] Monzel J.V., Budde T., Meyer Zu Schwabedissen H.E., Schwebe M., Bien-Möller S., Lütjohann D., Kroemer H.K., Jedlitschky G., Grube M. (2017). Doxorubicin enhances oxysterol levels resulting in a LXR-mediated upregulation of cardiac cholesterol transporters. Biochem. Pharmacol..

[75] Oram J.F., Vaughan A.M. (2000). ABCA1-mediated transport of cellular cholesterol and phospholipids to HDL apolipoproteins. Curr. Opin. Lipidol..

[76] Costet P., Luo Y., Wang N., Tall A.R. (2000). Sterol-dependent transactivation of the ABC1 promoter by the liver X receptor/retinoid X receptor. J. Biol. Chem..

[77] Qian H., Zhao X., Cao P., Lei J., Yan N., Gong X. (2017). Structure of the Human Lipid Exporter ABCA1. Cell.

[78] Fitzgerald M.L., Morris A.L., Chroni A., Mendez A.J., Zannis V.I., Freeman M.W. (2004). ABCA1 and amphipathic apolipoproteins form high-affinity molecular complexes required for cholesterol efflux. J. Lipid Res..

[79] Yamauchi Y., Hayashi M., Abe-Dohmae S., Yokoyama S. (2003). Apolipoprotein A-I activates protein kinase C alpha signaling to phosphorylate and stabilize ATP binding cassette transporter A1 for the high density lipoprotein assembly. J. Biol. Chem..

[80] Casteleijn M.G., Parkkila P., Viitala T., Koivuniemi A. (2018). Interaction of lecithin:cholesterol acyltransferase with lipid surfaces and apolipoprotein A-I-derived peptides. J. Lipid Res..

[81] Jonas A. (2000). Lecithin cholesterol acyltransferase. Biochim. Biophys. Acta.

[82] Gunawardane R.N., Fordstrom P., Piper D.E., Masterman S., Siu S., Liu D., Brown M., Lu M., Tang J., Zhang R. (2016). Agonistic Human Antibodies Binding to Lecithin-Cholesterol Acyltransferase Modulate High Density Lipoprotein Metabolism. J. Biol. Chem..

[83] Peelman F., Verschelde J.L., Vanloo B., Ampe C., Labeur C., Tavernier J., Vandekerckhove J., Rosseneu M. (1999). Effects of natural mutations in lecithin:cholesterol acyltransferase on the enzyme structure and activity. J. Lipid Res..

[84] Piper D.E., Romanow W.G., Gunawardane R.N., Fordstrom P., Masterman S., Pan O., Thibault S.T., Zhang R., Meininger D., Schwarz M. (2015). The high-resolution crystal structure of human LCAT. J. Lipid Res..

[85] Terasaka N., Wang N., Yvan-Charvet L., Tall A.R. (2007). High-density lipoprotein protects macrophages from oxidized low-density lipoprotein-induced apoptosis by promoting efflux of 7-ketocholesterol via ABCG1. Proc. Natl. Acad. Sci. USA.

[86] Li D., Zhang Y., Ma J., Ling W., Xia M. (2010). Adenosine monophosphate activated protein kinase regulates ABCG1-mediated oxysterol efflux from endothelial cells and protects against hypercholesterolemia-induced endothelial dysfunction. Arterioscler. Thromb. Vasc. Biol..

[87] Li J., Xiu Z., Wang R., Yu C., Chi Y., Qin J., Fu C., Matsuura E., Liu Q. (2017). The lipid moiety 7-ketocholesteryl-9-carboxynonanoate mediates binding interaction of oxLDL to LOX-1 and upregulates ABCA1 expression through PPARγ. Life Sci..

[88] Li J., Yu C., Wang R., Xu J., Chi Y., Qin J., Liu Q. (2017). The ω-carboxyl group of 7-ketocholesteryl-9-carboxynonanoate mediates the binding of oxLDL to CD36 receptor and enhances caveolin-1 expression in macrophages. Int. J. Biochem. Cell Biol..

[89] Wu A.L., Windmueller H.G. (1979). Relative contributions by liver and intestine to individual plasma apolipoproteins in the rat. J. Biol. Chem..

[90] Gerbod-Giannone M.C., Li Y., Holleboom A., Han S., Hsu L.C., Tabas I., Tall A.R. (2006). TNF-alpha induces ABCA1 through NF-kappaB in macrophages and in phagocytes ingesting apoptotic cells. Proc. Natl. Acad. Sci. USA.

[91] Shavva V.S., Mogilenko D.A., Nekrasova E.V., Trulioff A.S., Kudriavtsev I.V., Larionova E.E., Babina A.V., Dizhe E.B., Missyul B.V., Orlov S.V. (2018). Tumor necrosis factor α stimulates endogenous apolipoprotein A-I expression and secretion by human monocytes and macrophages: Role of MAP-kinases, NF-κB, and nuclear receptors PPARα and LXRs. Mol. Cell. Biochem..

[92] Suzuki K., Kawakami Y., Yamauchi K. (2017). Impact of TLR 2, TLR 4-activation on the Expression of ABCA1 and ABCG1 in Raw Cells. Ann. Clin. Lab. Sci..

[93] Takiguchi S., Ayaori M., Yakushiji E., Nishida T., Nakaya K., Sasaki M., Iizuka M., Uto-Kondo H., Terao Y., Yogo M. (2018). Hepatic Overexpression of Endothelial Lipase Lowers HDL (High-Density Lipoprotein) but Maintains Reverse Cholesterol Transport in Mice: Role of SR-BI (Scavenger Receptor Class B Type I)/ABCA1 (ATP-Binding Cassette Transporter A1)-Dependent Pathways. Arterioscler. Thromb. Vasc. Biol..

[94] Shrestha S., Wu B.J., Guiney L., Barter P.J., Rye K.A. (2018). Cholesteryl ester transfer protein and its inhibitors. J. Lipid Res..

[95] Masson D., Jiang X.C., Lagrost L., Tall A.R. (2009). The role of plasma lipid transfer proteins in lipoprotein metabolism and atherogenesis. J. Lipid Res..

[96] Beamer L.J., Carroll S.F., Eisenberg D. (1997). Crystal structure of human BPI and two bound phospholipids at 2.4 angstrom resolution. Science.

[97] Jiang X.C., Agellon L.B., Walsh A., Breslow J.L., Tall A. (1992). Dietary cholesterol increases transcription of the human cholesteryl ester transfer protein gene in transgenic mice. Dependence on natural flanking sequences. J. Clin. Investig..

[98] Martin L.J., Connelly P.W., Nancoo D., Wood N., Zhang Z.J., Maguire G., Quinet E., Tall A.R., Marcel Y.L., McPherson R. (1993). Cholesteryl ester transfer protein and high density lipoprotein responses to cholesterol feeding in men: Relationship to apolipoprotein E genotype. J. Lipid Res..

[99] Qiu X., Mistry A., Ammirati M.J., Chrunyk B.A., Clark R.W., Cong Y., Culp J.S., Danley D.E., Freeman T.B., Geoghegan K.F. (2007). Crystal structure of cholesteryl ester transfer protein reveals a long tunnel and four bound lipid molecules. Nat. Struct. Mol. Biol..

[100] Li T., Chiang J.Y. (2015). Bile acids as metabolic regulators. Curr. Opin. Gastroenterol..

[101] Russell D.W. (2003). The enzymes, regulation, and genetics of bile acid synthesis. Annu. Rev. Biochem..

[102] Li-Hawkins J., Gåfvels M., Olin M., Lund E.G., Andersson U., Schuster G., Björkhem I., Russell D.W., Eggertsen G. (2002). Cholic acid mediates negative feedback regulation of bile acid synthesis in mice. J. Clin. Investig..

[103] Chiang J.Y. (2009). Bile acids: Regulation of synthesis. J. Lipid Res..

[104] Gupta S., Pandak W.M., Hylemon P.B. (2002). LXR alpha is the dominant regulator of CYP7A1 transcription. Biochem. Biophys. Res. Commun..

[105] Davis R.A., Miyake J.H., Hui T.Y., Spann N.J. (2002). Regulation of cholesterol-7alpha-hydroxylase: BAREly missing a SHP. J. Lipid Res..

[106] Tarling E.J., Clifford B.L., Cheng J., Morand P., Cheng A., Lester E., Sallam T., Turner M., de Aguiar Vallim T.Q. (2017). RNA-binding protein ZFP36L1 maintains posttranscriptional regulation of bile acid metabolism. J. Clin. Investig..

[107] Goodwin B., Jones S.A., Price R.R., Watson M.A., McKee D.D., Moore L.B., Galardi C., Wilson J.G., Lewis M.C., Roth M.E. (2000). A regulatory cascade of the nuclear receptors FXR, SHP-1, and LRH-1 represses bile acid biosynthesis. Mol. Cell.

[108] Reeskamp L.F., Meessen E.C.E., Groen A.K. (2018). Transintestinal cholesterol excretion in humans. Curr. Opin. Lipidol..

[109] Le May C., Berger J.M., Lespine A.L., Pillot B., Prieur X., Letessier E., Hussain M.M., Collet X., Cariou B., Costet P. (2013). Transintestinal cholesterol excretion is an active metabolic process modulated by PCSK9 and statin involving ABCB1. Arterioscler. Thromb. Vasc. Biol..

[110] Vrins C.L., Ottenhoff R., van den Oever K., de Waart D.R., Kruyt J.K., Zhao Y., van Berkel T.J., Havekes L.M., Aerts J.M., van Eck M. (2012). Trans-intestinal cholesterol efflux is not mediated through high density lipoprotein. J. Lipid Res..

[111] Van der Velde A.E., Vrins C.L., van den Oever K., Seemann I., Oude Elferink R.P., van Eck M., Kuipers F., Groen A.K. (2008). Regulation of direct transintestinal cholesterol excretion in mice. Am. J. Physiol. Gastrointest. Liver Physiol..

[112] Talbot C.P.J., Plat J., Ritsch A., Mensink R.P. (2018). Determinants of cholesterol efflux capacity in humans. Prog. Lipid Res..

[113] McGillicuddy F.C., de la Llera Moya M., Hinkle C.C., Joshi M.R., Chiquoine E.H., Billheimer J.T., Rothblat G.H., Reilly M.P. (2009). Inflammation impairs reverse cholesterol transport in vivo. Circulation.

[114] O’Reilly M., Dillon E., Guo W., Finucane O., McMorrow A., Murphy A., Lyons C., Jones D., Ryan M., Gibney M. (2016). High-Density Lipoprotein Proteomic Composition, and not Efflux Capacity, Reflects Differential Modulation of Reverse Cholesterol Transport by Saturated and Monounsaturated Fat Diets. Circulation.

[115] Castilho G., Okuda L.S., Pinto R.S., Iborra R.T., Nakandakare E.R., Santos C.X., Laurindo F.R., Passarelli M. (2012). ER stress is associated with reduced ABCA-1 protein levels in macrophages treated with advanced glycated albumin—Reversal by a chemical chaperone. Int. J. Biochem. Cell Biol..

[116] De Souza Pinto R., Castilho G., Paim B.A., Machado-Lima A., Inada N.M., Nakandakare E.R., Vercesi A.E., Passarelli M. (2012). Inhibition of macrophage oxidative stress prevents the reduction of ABCA-1 transporter induced by advanced glycated albumin. Lipids.

[117] Zhou Z., Tang Y., Jin X., Chen C., Lu Y., Liu L., Shen C. (2016). Metformin Inhibits Advanced Glycation End Products-Induced Inflammatory Response in Murine Macrophages Partly through AMPK Activation and RAGE/NFκB Pathway Suppression. J. Diabetes Res..

[118] Iborra R.T., Machado-Lima A., Okuda L.S., Pinto P.R., Nakandakare E.R., Machado U.F., Correa-Giannella M.L., Pickford R., Woods T., Brimble M.A. (2018). AGE-albumin enhances ABCA1 degradation by ubiquitin-proteasome and lysosomal pathways in macrophages. J. Diabetes Complicat..

[119] Daffu G., Shen X., Senatus L., Thiagarajan D., Abedini A., Hurtado Del Pozo C., Rosario R., Song F., Friedman R.A., Ramasamy R. (2015). RAGE Suppresses ABCG1-Mediated Macrophage Cholesterol Efflux in Diabetes. Diabetes.

[120] Aleidi S.M., Howe V., Sharpe L.J., Yang A., Rao G., Brown A.J., Gelissen I.C. (2015). The E3 ubiquitin ligases, HUWE1 and NEDD4-1, are involved in the post-translational regulation of the ABCG1 and ABCG4 lipid transporters. J. Biol. Chem..

[121] Aleidi S.M., Yang A., Sharpe L.J., Rao G., Cochran B.J., Rye K.A., Kockx M., Brown A.J., Gelissen I.C. (2018). The E3 ubiquitin ligase, HECTD1, is involved in ABCA1-mediated cholesterol export from macrophages. Biochim. Biophys. Acta.

[122] Clayton Z.S., Fusco E., Kern M. (2017). Egg consumption and heart health: A review. Nutrition.

[123] US Department of Health and Human Services, US Department of Agriculture (2015). 2015–2020 Dietary Guidelines for Americans.

[124] Virtanen J.K., Mursu J., Virtanen H.E., Fogelholm M., Salonen J.T., Koskinen T.T., Voutilainen S., Tuomainen T.P. (2016). Associations of egg and cholesterol intakes with carotid intima-media thickness and risk of incident coronary artery disease according to apolipoprotein E phenotype in men: The Kuopio Ischaemic Heart Disease Risk Factor Study. Am. J. Clin. Nutr..

[125] Goldberg S., Gardener H., Tiozzo E., Ying Kuen C., Elkind M.S., Sacco R.L., Rundek T. (2014). Egg consumption and carotid atherosclerosis in the Northern Manhattan study. Atherosclerosis.

[126] Rhee E.J., Ryu S., Lee J.Y., Lee S.H., Cheong E., Park S.E., Park C.Y., Won Y.S., Kim J.M., Cho D.S. (2017). The association between dietary cholesterol intake and subclinical atherosclerosis in Korean adults: The Kangbuk Samsung Health Study. J. Clin. Lipidol..

[127] Choi Y., Chang Y., Lee J.E., Chun S., Cho J., Sung E., Suh B.S., Rampal S., Zhao D., Zhang Y. (2015). Egg consumption and coronary artery calcification in asymptomatic men and women. Atherosclerosis.

[128] Blesso C.N., Fernandez M.L. (2018). Dietary Cholesterol, Serum Lipids, and Heart Disease: Are Eggs Working for or Against You?. Nutrients.

[129] Shin J.Y., Xun P., Nakamura Y., He K. (2013). Egg consumption in relation to risk of cardiovascular disease and diabetes: A systematic review and meta-analysis. Am. J. Clin. Nutr..

[130] Barraj L., Tran N., Mink P. (2009). A comparison of egg consumption with other modifiable coronary heart disease lifestyle risk factors: A relative risk apportionment study. Risk Anal..

[131] Herron K.L., Vega-Lopez S., Conde K., Ramjiganesh T., Shachter N.S., Fernandez M.L. (2003). Men classified as hypo- or hyperresponders to dietary cholesterol feeding exhibit differences in lipoprotein metabolism. J. Nutr..

[132] García-Otín A.L., Cofán M., Junyent M., Recalde D., Cenarro A., Pocoví M., Ros E., Civeira F. (2007). Increased intestinal cholesterol absorption in autosomal dominant hypercholesterolemia and no mutations in the low-density lipoprotein receptor or apolipoprotein B genes. J. Clin. Endocrinol. Metab..

[133] Grundy S.M. (2016). Does Dietary Cholesterol Matter?. Curr. Atheroscler. Rep..

[134] Ravera A., Carubelli V., Sciatti E., Bonadei I., Gorga E., Cani D., Vizzardi E., Metra M., Lombardi C. (2016). Nutrition and Cardiovascular Disease: Finding the Perfect Recipe for Cardiovascular Health. Nutrients.

